# P2Y_2_ Inhibition Modifies the Anabolic Response to Exercise in Adult Mice

**DOI:** 10.1111/acel.14464

**Published:** 2024-12-31

**Authors:** Amit Chougule, Chunbin Zhang, Jordan Denbow, Nickolas Vinokurov, Devin Mendez, Elizabeth Vojtisek, Joseph Gardinier

**Affiliations:** ^1^ Bone and Joint Center Henry Ford Health System Detroit Michigan USA; ^2^ Henry Ford Health + Michigan State University Health Sciences Detroit Michigan USA; ^3^ Department of Physiology College of Human Medicine Michigan State University East Lansing Michigan USA; ^4^ School of Medicine Wayne State University Detroit Michigan USA

**Keywords:** mechanobiology, osteoporosis, purinergic signaling

## Abstract

As the aging population continues to grow, the incidence of osteoporotic fractures increases and is compounded by our lack of therapeutic strategies that increase bone formation. Although exercise and physical activity play a key role in maintaining bone mass throughout our lives, the loads and exertion required to elicit an anabolic response becomes exceedingly difficult to achieve with age. Based on previous work, the P2Y_2_ receptor offers a unique therapeutic target to increasing bone mass by modifying the mechanotransduction. Others have also shown P2Y_2_ to have a negative effect on osteoblast function. However, the extent to which inhibiting P2Y_2_ pharmaceutically improves bone mass or the mechanotransduction of bone remains unknown. Our central hypothesis for this study states that inhibiting P2Y_2_ activity can enhance the anabolic response to loading in an aging population. To test this hypothesis, the anabolic response to exercise was examined by treating adult mice, which typically display a minimal response, with the P2Y_2_ inhibitor AR‐C118925XX (ARC). Our findings from this study demonstrate that ARC treatment of adult mice increases periosteal bone formation in response to exercise. The enhanced response to exercise was characterized by a reduction in osteocytes' induction of osteoclast activity. Endocortical bone formation also increased with treatment independently of exercise, providing gains in mechanical strength and tissue level properties. Overall, inhibiting P2Y_2_ activation has a beneficial effect on bone formation and the anabolic response to loading, namely by limiting osteoclast activation.

## Graphical abstract

Inhibition of the P2Y2 receptor with AR‐C118925XX (ARC) inhibits osteocytes induction of osteoclast differentiation. Treating adult mice with ARC prevents age‐related bone loss and increase endocortical bone formation. At the same time, ARC also enhances the anabolic response to exercise in aged mice by increasing the degree of periosteal bone formation.

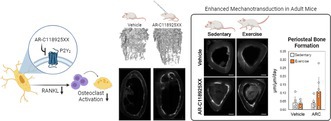

## 1 Introduction

Age‐related bone loss predisposes adults to osteoporosis and fracture risk. The growing incidence and economic burden of osteoporotic fractures is compounded by the lack of therapeutic strategies that promote bone formation or improve tissue strength. Skeletal loading plays a critical role in maintaining bone mass, such that exercise is considered a preventative measure against osteoporosis (Karl Karlsson and Erik Rosengren 2012). The induction of bone formation in response to loading is extremely age dependent, such that clinical studies have shown exercise to be less and less effective with age (Nguyen, Center, and Eisman 2000; Marques, Mota, and Carvalho 2012). In vivo loading models have gone on to demonstrate a lack of responsiveness in aged animals (Holguin, Brodt, and Silva 2016; Gardinier et al. 2018; Turner, Takano, and Owan 1995; Srinivasan, Gross, and Bain 2012; Rubin, Bain, and McLeod 1992). The lack of response is readily attributed to a shift in the mechanosensitivity of osteocytes, the primary cell responsible for the mechanotransduction of bone (Holguin, Brodt, and Silva 2016). As a result, the loads and exertion often required to elicit osteocytes' response or increase bone mass can be difficult to achieve in an aging population. Targeting the underlining pathways that regulate the mechanosensitivity of bone offer an alternative strategy to increasing bone mass under lower loads or during daily activities that are more amendable to an aging population.

Purinergic signaling plays a key role in the mechanotransduction of bone and overall maintenance of bone mass by regulating an array of downstream signaling pathways through specific purinergic receptors (Orriss, Burnstock, and Arnett 2010). In particular, purinergic signaling through the P2X_7_ receptor facilitates the anabolic response to loading by activating Wnt/β‐catenin signaling pathways (Ke et al. 2003; Li et al. 2005; Grol et al. 2016). However, developing clinical strategies that mimic the anabolic effects of P2X_7_ through specific agonists has proven extremely difficult considering that P2X_7_ activation among osteoblasts inhibits mineralization (Orriss et al. 2012). Of the various P2Y subtypes, the P2Y_2_ receptor in particular is the most abundantly expressed by osteocytes (Orriss et al. 2012). Global knockout models of the P2Y receptor exhibit increased bone mass, which has been attributed to enhanced osteoblast function or mechanotransduction (Orriss, Burnstock, and Arnett 2010; Orriss et al. 2011; Gardinier et al. 2014). As a result, P2Y_2_ represents a novel therapeutic target with the potential for enhancing the mechanotransduction of bone and increasing overall bone mass.

Over the years extensive work has gone into developing selective antagonists targeting P2Y_2_ for the purpose of preventing tumor metastasis, skin fibrosis, cystic fibrosis, inflammation, and atherosclerosis (Burnstock 2017). Currently the most selective antagonist is the compound AR‐C118925 (Rafehi et al. 2017). Animal studies have found AR‐C118925 to alleviate chronic pain, pancreatic cancer metastasis, skin fibrosis, as well as ischemic heart disease and atherosclerosis (Chen et al. 2017; Perera et al. 2018; Hu et al. 2018; Magni et al. 2015). However, the impact of AR‐C118925 on bone formation and preventing age‐related bone loss is entirely unknown. Our central hypothesis states that inhibiting P2Y_2_ activity can enhance the anabolic response to loading. Here we describe the anabolic effects of exercise and daily loading when treating adult mice with the P2Y_2_ inhibitor AR‐C118925XX (ARC). In addition, we used in vitro studies to examine the direct and indirect effects of ARC on osteoblast and osteoclast behavior.

## 2 Results

### 2.1 P2Y_2_ Inhibition in Adult Mice Enhances Periosteal Expansion in Response to Exercise

**FIGURE 1:**
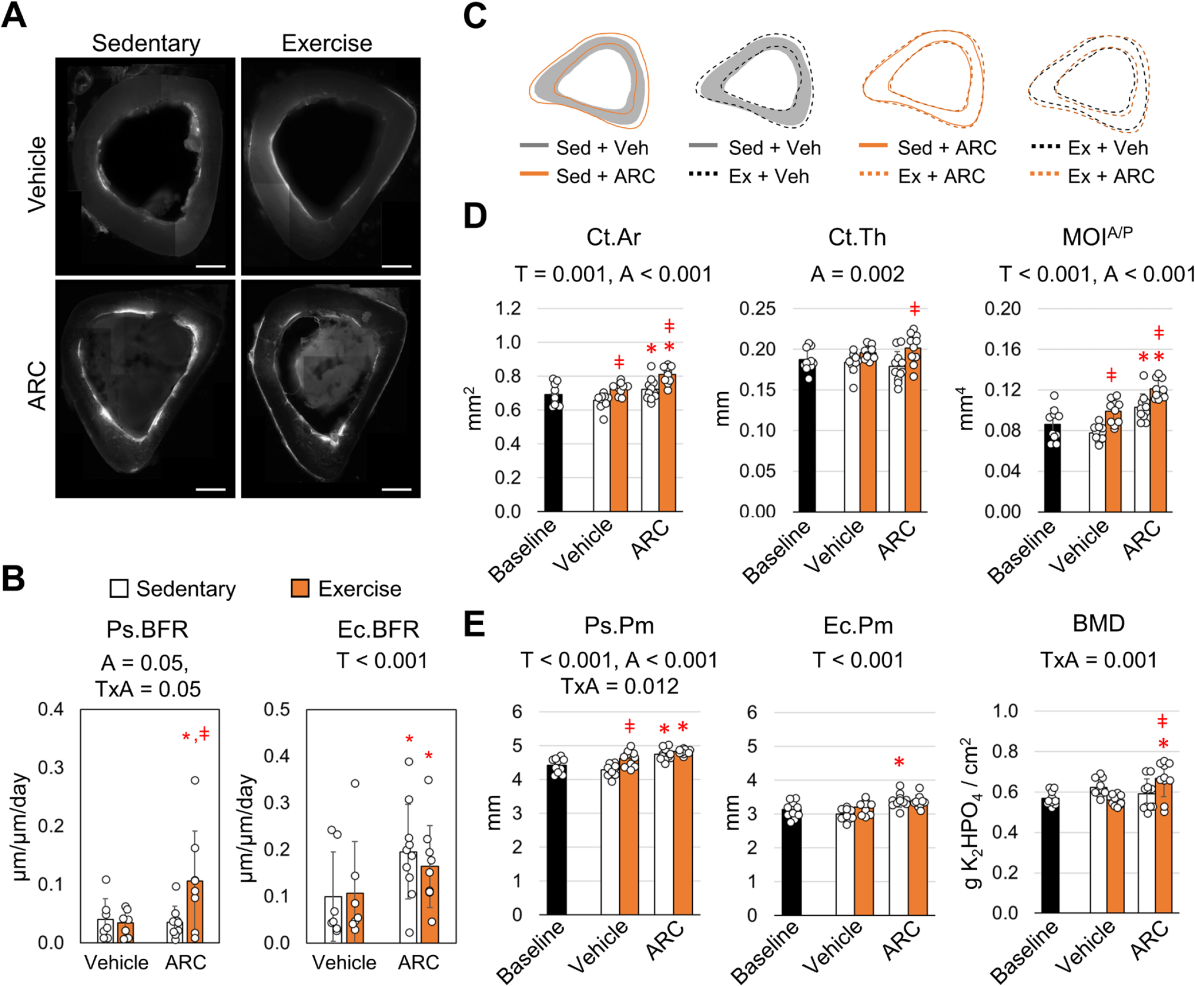
AR‐C118925XX treatment of adult mice enhances periosteal bone formation in response to exercise. (A) Representative images of double labeling at the mid‐diaphysis of the tibia (bar represents 100 μm). (B) Periosteal bone formation (Ps.BFR) and endocortical bone formation rate (Ec.BFR) were measured based on histomorphometry. (C) Traces of the periosteal and endocortical surfaces for each group are overlaid to show comparisons between groups. (D–E) Micro‐CT analysis was used to quantify cortical bone area (Ct.Ar), thickness (Ct.Th), moment of inertia about the anterior/posterior axis (MOI^A/P^), periosteal perimeter (Ps.Pm), endocortical perimeter (Ec.Pm), and bone mineral density (BMD). Two‐way ANOVA identified main effects for treatment (T), activity (A), and their interaction (T × A). Tukey post hoc analysis identified significant differences between groups (*p < 0.05 compared to vehicle control, ǂ *p* < 0.05 compared to sedentary control). Bars show mean ± std. (*n* = 9, data points represent individual mice).

Purinergic signaling through the P2Y_2_ receptor is known to negatively impact bone mass and the mechanotransduction of bone cells. To understand how inhibiting P2Y_2_ can enhance bone formation in an aging population, male mice were treated with AR‐C118925XX (ARC) for 5‐weeks while also being subjected to treadmill exercise. No significant differences in body weight were observed between each group after 5‐weeks of their respective treatments (Table S1). The anabolic response to treadmill exercise in 35‐week old mice is markedly smaller compared to 8‐week mice, which are not skeletally mature (Gardinier et al. 2018). Similar to our previous work, periosteal bone formation rate (Ps.BFR) did not increase with exercise in vehicle treated controls (Figure 1A,B). Conversely, ARC treatment prior to exercise significantly increased Ps.BFR by 3‐fold when compared to ARC treated sedentary controls as well as vehicle treated exercise mice. The increased bone formation was mediated by a significant increase in periosteal mineralization (Ps.MS/BS), while the mineral apposition rate (Ps.MAR) remained the same (Table S2). These findings indicate that the quantity of tissue osteoblast form is increased, but the rate at which osteoblast form the tissue or facilitate mineralization remains the same.

Similar to our previous exercise models, endocortical indices were largely unaffected by exercise across both vehicle and ARC treated groups (Gardinier et al. 2018; Gardinier, Mohamed, and Kohn 2015). However, ARC treatment across both sedentary and exercise groups significantly increased the endocortical bone formation rate (Ec.BFR) by 75% compared to vehicle treated mice. The increase in endocortical bone formation was mediated by an increase in both endocortical mineralization (Ec.MS/BS) as well as mineral apposition rate (Ec.MAR) (Table S2), suggesting that not only are osteoblasts' forming more tissue, but also at a higher rate in the presence of ARC.

The gains in periosteal and endocortical bone formation mediated by ARC and exercise were coupled with changes in the cortical geometry, which were evident by mapping the average periosteal and endocortical perimeters (Figure 1C) of the tibea. In particular, ARC treatment was shown to increase cortical area (Ct. Ar), which increased further with the addition of exercise (Figure 1D). The addition of exercise with ARC treatment also significantly increased cortical thickness (Ct.Th) by 13% compared to ARC treatment alone (Figure 1D). In contrast, exercise among vehicle treated mice failed to produce a similar effect. Furthermore, exercise with ARC significantly increased the moment of inertia about the anterior/posterior (MOI^A/P^) axis by 13% compared to ARC treated mice alone (Figure 1D). For vehicle treated mice, the increase in MOI following exercise was significantly less. The increase in MOI as a result of ARC treatment corresponded with an increase in both expansion at the periosteal perimeter (Ps.Pm) and endocortical perimeter (Ec.Pm) (Figure 1E). The Ps.Pm for sedentary mice treated with ARC exhibited a 10.5% increase while the Ec.Pm displayed a 13.5% increase compared to vehicle controls. Although the addition of exercise with ARC did not increase Ps.Pm further, the Ps.Pm was 4.3% greater than exercise with vehicle. Exercise had no effect on BMD in vehicle treated mice (Figure 1E). However, ARC with exercise increased BMD by 11%, suggesting ARC has an additive effect on BMD and cortical architecture during exercise.

### 2.2 P2Y_2_ Inhibition in Adult Mice Enhances Mechanical Strength of Bone

**FIGURE 2:**
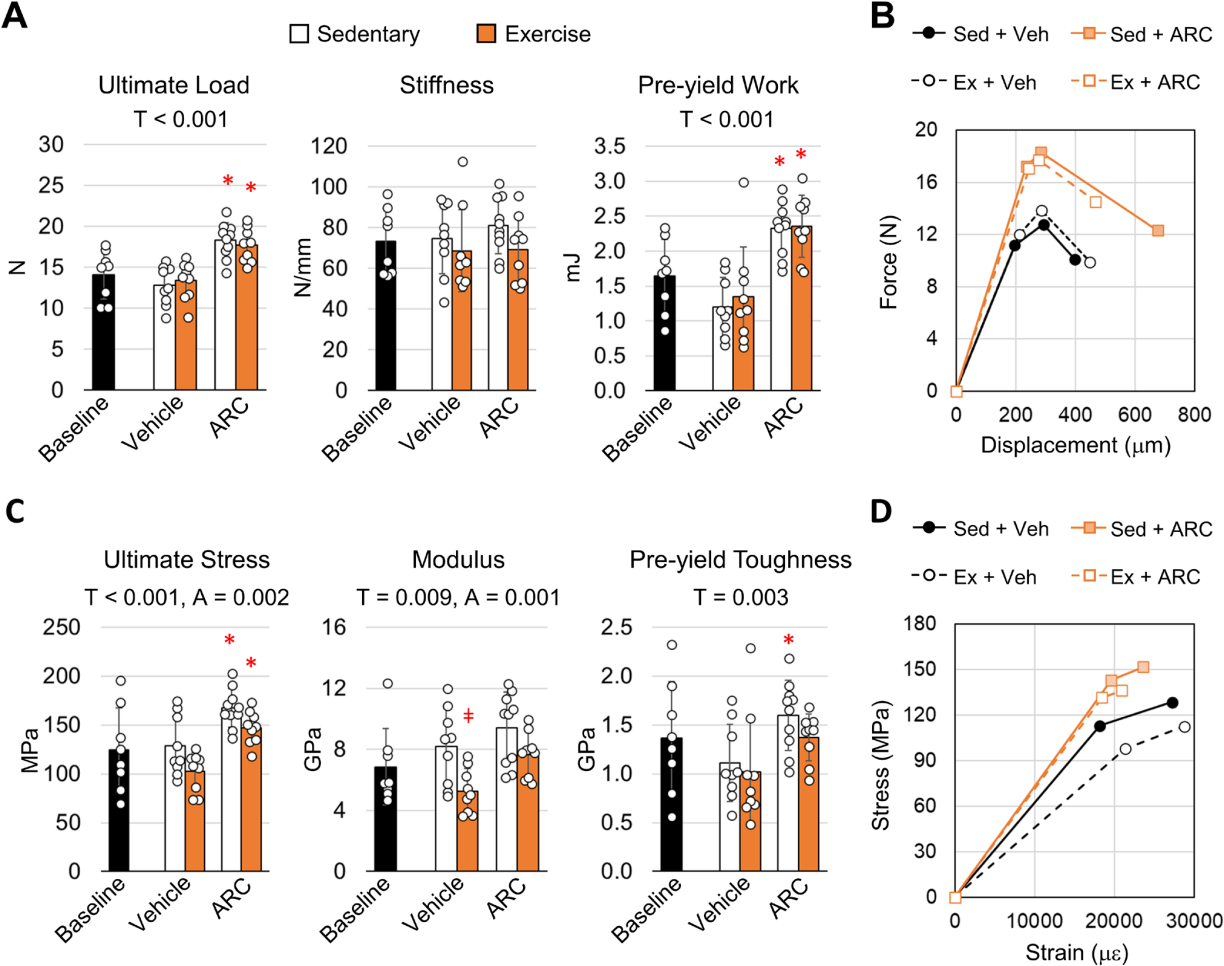
AR‐C118925XX treatment of adult mice increases bone strength and tissue properties independent of exercise. (A) The structural‐level properties ultimate load, stiffness, and pre‐yield work were measured under four‐point bending. (B) Average force–deflection curve for the tibea of sedentary mice treated with vehicle control or AR‐118925. (C) The tissue‐level properties ultimate stress, modulus, and pre‐yield work were calculated. (D) Average stress–strain curve calculated for the tibia of sedentary mice treated with vehicle control or AR‐118925. Two‐way ANOVA identified main effects for treatment (T), activity (A), and their interaction (T × A). Tukey post hoc analysis identified significant differences between groups (*p < 0.05 compared to vehicle control, ǂ *p* < 0.05 compared to sedentary control). Bars show mean ± std. (*n* = 9, data points represent individual mice).

To understand how bone strength was then impacted by the response to ARC and exercise, the mechanical properties of the tibia were measured under four‐point bending. Similar to our previous studies, vehicle treated mice failed to exhibit any significant changes in the structural‐level properties ultimate load, stiffness, and pre‐yield work following treadmill exercise (Figure 2A) (Gardinier et al. 2018). Exercise in vehicle treated mice also produced an increase in the post‐yield properties (Table S3), similar to that seen in younger mice (Wallace, Ron, and Kohn 2009; Wallace et al. 2010). However, the corresponding increase in cortical area and MOI following exercise (Figure 1C) suggests a loss in tissue‐level properties, which is evident by the significant difference in modulus between vehicle treated sedentary and exercise groups (Figure 2D). In contrast, the increase in bone mass following ARC treatment and exercise was matched with a significant increase in the ultimate load along with an increase in ultimate stress, suggesting an increase in tissue‐level properties. The ARC treated mice also exhibited a significant increase in the pre‐yield work and pre‐yield toughness, both of which represent the area under the force‐displacement and stress‐strain curves respectively (Figure 2C,D). These findings suggest an increase in the general capacity for bone to absorb energy under loading following ARC treatment. Despite added gains in cortical area and MOI, the addition of ARC treatment with exercise failed to have any additive effect on bone strength compared to ARC treatment alone.

### 2.3 P2Y_2_ Inhibition Enhances Endocortical Bone Formation Independent of Exercise

**FIGURE 3:**
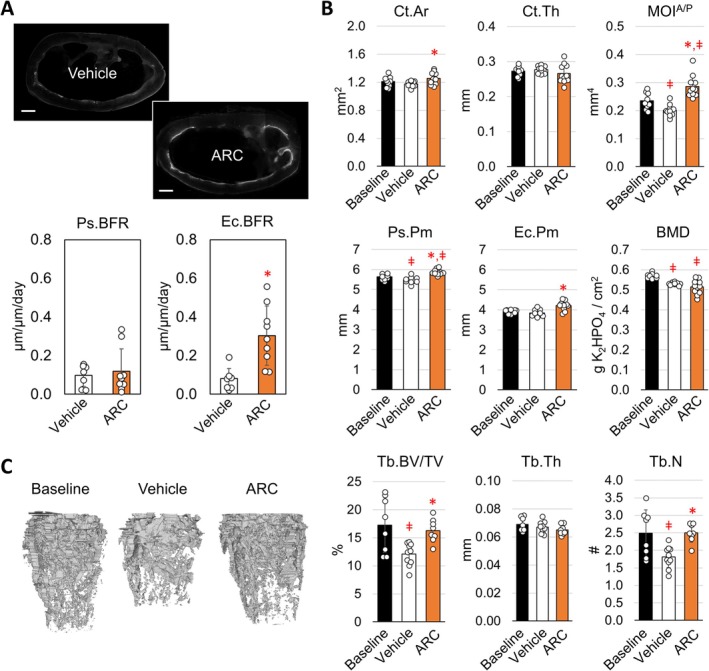
AR‐C118925XXX prevents bone loss and increases endocortical bone formation in the femur of sedentary adult mice. (A) Representative images of double labeling at the mid‐diaphysis of the femur (bar represents 100 μm), and quantification of the periosteal bone formation (Ps.BFR) and endocortical bone formation rate (Ec.BFR). (B) Quantification of the cortical bone area (Ct.Ar), thickness (Ct.Th), moment of inertia about the anterior/posterior axis (MOI^A/P^), periosteal perimeter (Ps.Pm), endocortical perimeter (Ec.Pm), and bone mineral density (BMD) of the femur. (C) Representative images and quantification of the distal femoral trabecular bone volume (Tb.BV/TV), thickness (Tb.Th), and number (Tb.N). A one‐way ANOVA and Tukey post hoc analysis identified significant differences between groups (*p < 0.05 compared to vehicle control, ǂ *p* < 0.05 compared to baseline). Bars show mean ± std. (*n* = 9, data points represent individual mice).

Given that ARC treatment alone has such a significant impact independent of exercise, we then examined if the effects of ARC are isolated to the tibia or recapitulated in other long bones. Similar to what was observed for the tibia, the femur exhibited greater fluorochrome labeling at the endocortical surface (Figure 3A). A significant increase in Ec.BFR was observed in response to ARC treatment, which was mediated through an increase in the Ec.MS/BS (Table S4). At the same time, ARC treated mice exhibited significantly greater Ct. Ar and MOI compared to vehicle treated controls (Figure 3B), while the gains in MOI in particular were also significantly greater than baseline by 4%. In contrast, vehicle treated mice displayed a significant decrease in the Ps.Pm compared to baseline, which also corresponded with a decrease in Ct.Ar and MOI. The decrease in bone mass suggests a significant degree of resorption under sedentary conditions, located largely at the periosteal surface. Despite the differences in cortical geometry, both vehicle and ARC treated mice exhibited a loss in BMD over the course of the experiment.

The loss in cortical bone under sedentary conditions coincided with a significant loss in trabecular bone in the distal femur (Figure 3C). Both trabecular bone volume (Tb.BV/TV) and trabecular number (Tb.N) decreased by 30% and 27% respectively from baseline. The connectivity density (Conn.D) and degree of anisotropy (Tb.DA) also significantly decreased in vehicle treated sedentary mice by 42% and 13% respectively (Table S5). ARC treatment completely mitigated the decrease in Tb.BV/TV, Tb.N, Conn.D, and Tb.DA. At the same time, ARC treated mice exhibited a significant decrease in the structural model index (Table S5), indicating a more plate‐like structure as opposed to the rod‐like structure. Altogether these findings suggest that ARC treatment prevents bone loss while normal levels of bone formation are maintained.

### 2.4 ARC Modifies Osteocytes' Gene Expression and Induction of Osteoclast Activation

**FIGURE 4:**
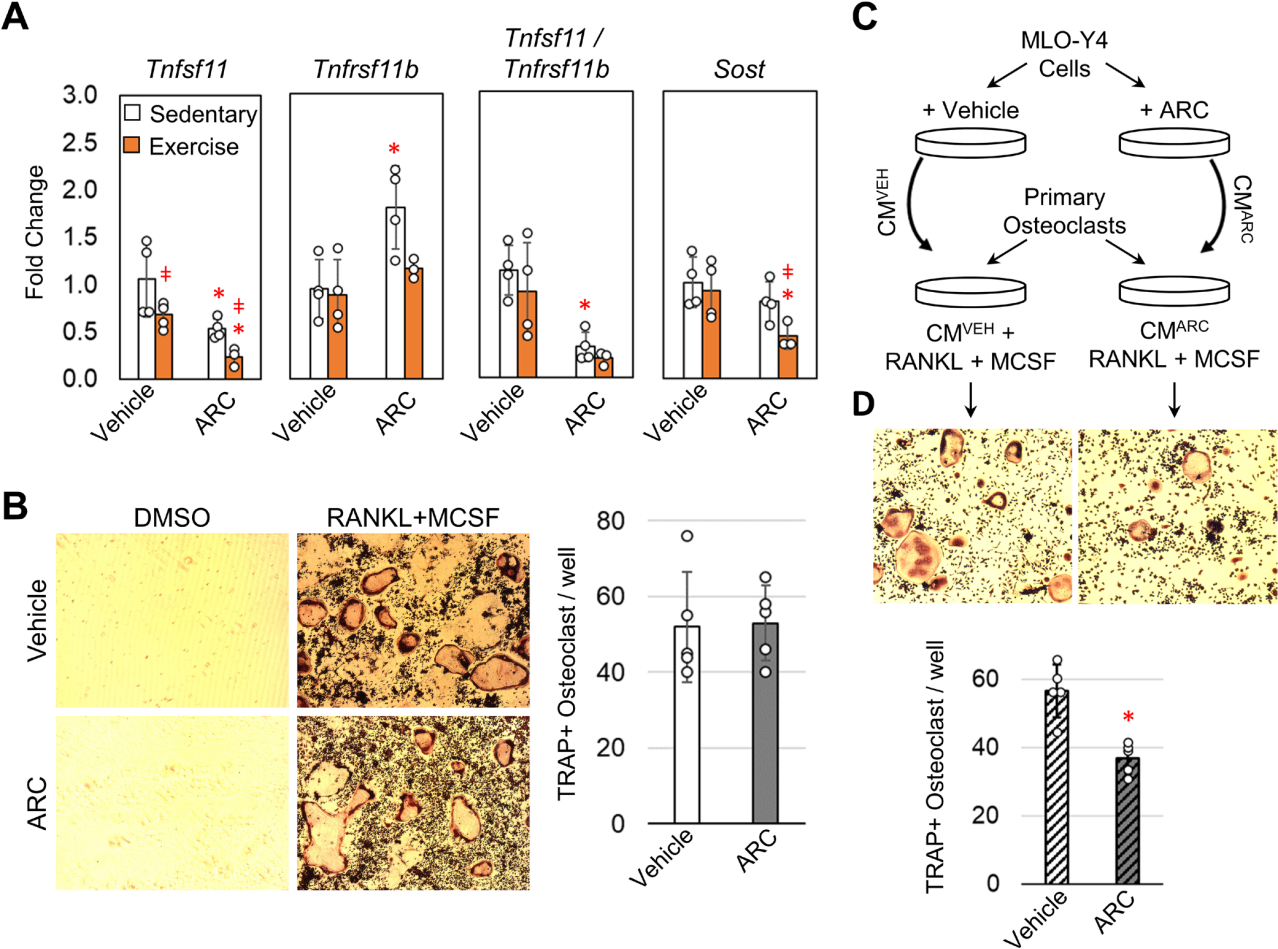
AR‐C118925XXX reduces osteocytes' support of osteoclastogenesis. (A) Osteocyte‐enriched cortical bone samples were used to measure mRNA expression in the tibia of mice following 1‐week of exercise or sedentary conditions alongside vehicle or ARC treatment. Student *t*‐ttest identified significant differences between groups (*p < 0.05 compared to vehicle control, ǂ *p* < 0.05 compared to sedentary control). Bars show means ± std. (*n* = 3–4, data points represent individual mice). (B) Representative images of tartrate‐resistant acid phosphatase positive (TRAP+) osteoclasts following 7 days of differentiation in the presence of DMSO or ARC (10 μM) Bars show mean ± std. (*n* = 5, data points represent biological replicates). (C) Conditioned media from MLO‐Y4 cells treated with DMS or ARC (10 μM) was applied to primary osteoclast cultures. (D) Representative images and quantification of TRAP+ osteoclasts following differentiation in the presence of RANKL and MCSF. Student *t*‐test identified significant differences between groups (*p < 0.05 compared to vehicle control). Bars show mean ± std. (*n* = 4, data points represent biological replicates).

Because the induction of bone formation and bone resorption are tightly regulated by osteocytes' response to loading, osteocyte gene expression was evaluated following 1‐week of ARC treatment and exercise by isolating mRNA from osteocyte‐enriched cortical bone samples. The initial response to exercise was characterized by a significant decrease in *Tnfsf11* (encodes Rankl), while *Tnfrsf11b* (encodes Opg) and *Sost* expression were largely unaffected (Figure 4A). The lack of an effect on *Sost* expression is consistent across other loading models of aged mice (Holguin, Brodt, and Silva 2016; Gardinier et al. 2018). For ARC treated mice, both *Tnfsf11* and *Sost* expression were significantly reduced with the addition of exercise and ARC treatment. ARC treatment in sedentary mice also increased *Tnfrsf11b* expression significantly by 0.8‐fold. However, the ratio between *Tnfsf11* to *Tnfrsf11b* was significantly smaller in ARC treated sedentary mice compared to vehicle treated sedentary mice. These data suggest a decrease in osteoclast behavior that would coincide with the lack of bone loss observed after 5‐weeks of treatment.

Given that ARC treatment alone was able to suppress the Tnfsf11/Tnfrsf11b ratio independent of exercise, we further examined the effect of ARC on osteoclast activation by first differentiating primary hematopoietic stem cells in the presence of ARC. In the presence of RANKL and MCSF osteoclast differentiation is noted by the increase in tartrate‐resistant acid phosphatase (TRAP) (Figure 4B) compared to DMSO treated controls. The number of TRAP positive cells was largely unaffected by the addition of ARC during the differentiation, suggesting that direct inhibition of the P2Y_2_ receptor in osteoclast precursors does not prevent activation. To further test if there is an indirect effect of ARC, the media used to differentiate osteoclasts was supplemented with conditioned media from MLO‐Y4 cells that had been treated with ARC (10 μM) or DMSO as a vehicle control (Figure 4C). The conditioned media from vehicle treated MLO‐Y4 cells supported osteoclast activation based on the induction of multinucleated TRAP positive cells (Figure 4D). Conversely, the conditioned media from ARC treated MLO‐Y4 cells had a significantly reduced effect on osteoclast activation based on the number of TRAP positive cells. These data suggest that ARC can limit osteoclast activation mediated by osteocytes.

### 2.5 ARC Treatment Modifies Osteoblasts Expression Profile of Matrix Proteins

**FIGURE 5:**
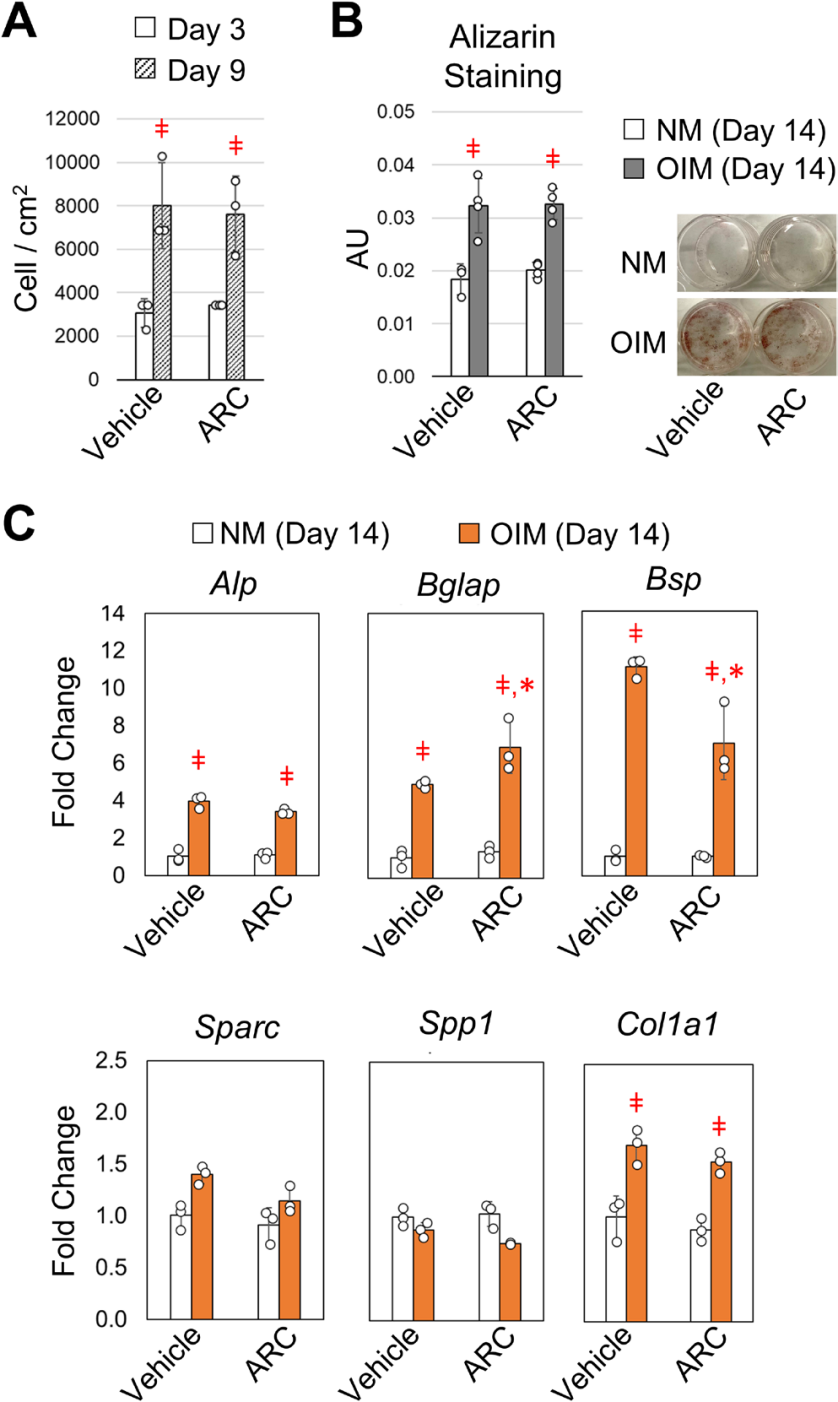
Osteoblast growth and mineralization are uninhibited by AR‐C118925XXX, but exhibit a shift in expression of non‐collagenous proteins. (A) The number of primary bone marrow stromal cells were quantified following 3 and 9 days of differentiation in media supplemented with vehicle or ARC (10 μM). Student *t*‐test identified significant differences between time points (ǂ *p* < 0.05 compared to Day 3). (B) Representative images and quantification of alizarin red staining following 10 days of culture in normal media (NM) or osteogenic inducing media (OIM) supplemented with vehicle or ARC (10 μM). Student *t*‐test identified significant differences between type of media (ǂ *p* < 0.05 compared to NM). (C) The mRNA expression of type‐1 collagen 1a (*Col1a1*), alkaline phosphate (*Alp*), bone sialoprotein (*Bsp*), osteocalcin (*Bglap*), osteonectin (*Sparc*), and osteopontin (*Opn*) were measured using qRT‐PCR. Student *t*‐test identified significant differences between time points and treatments (ǂ *p* < 0.05 compared to Day 3, *p < 0.05 compared to vehicle control). Bars show mean ± std. (*n* = 3, data points represent biological replicates).

The increase in bone formation and tissue‐level mechanical properties suggest a shift in osteoblast behavior or activity following ARC treatment. To further understand how ARC effects osteoblast function, bone marrow stromal cells from adult mice were treated with osteogenic inducing media (OIM) that was supplemented with ARC (10 μM) or vehicle control. Based on cell numbers, osteoblast growth over the course of 6 days was the same when cultured in the presence of ARC or vehicle control (Figure 5A). The degree of mineralization after 14 days of culture was also the same for ARC and vehicle treated cells based on alizarin red staining (Figure 5B). We then examined changes in gene expression related to extracellular matrix proteins that contribute to the tissue properties of bone. After 14 days of differentiation, gene expression for type‐1a collagen (*Col1a1*) and alkaline phosphate (*Alp*) was significantly increased and displayed a similar level of expression with or without ARC present in the media (Figure 5C). The expression of bone sialoprotein (*Bsp*) and osteocalcin (*Bglap*) increased with differentiation; however, *Bsp* expression was significantly less in the presence of ARC, while osteocalcin expression was significantly greater in the presence of ARC compared to vehicle controls. Other matrix proteins, namely osteonectin (*Sparc*) and osteopontin (*Spp1*) displayed minimal changes. Altogether, these data demonstrate a shift in the expression of matrix proteins that play a key role in regulating mineralization and the tissue properties of bone.

## 3 Discussion

The P2Y_2_ receptor has been considered as a potential target for increasing bone formation by enhancing mechanotransduction as well as osteoblast function. The small molecule inhibitor AR‐C118925XX (ARC) allowed us to test the hypothesis that inhibiting P2Y_2_ enhances the mechanotransduction of bone and prevents bone loss in an aging population. In support of our hypothesis, ARC treatment enhanced the response treadmill exercise based on gains in periosteal bone formation and periosteal expansion, which are characteristic of the anabolic response to loading. Across aged animal models the periosteal response to loading is markedly diminished (Holguin, Brodt, and Silva 2016; Turner, Takano, and Owan 1995; Srinivasan, Gross, and Bain 2012; Rubin, Bain, and McLeod 1992). Clinical studies have gone on to find exercise to have the largest effect during the pre‐pubertal period, while adults exhibit minimal gains in bone mass and BMD (Karl Karlsson and Erik Rosengren 2012). By using 9‐month old mice, we can assume the lack of response to exercise is a function of changes in osteocyte mechanobiology or down‐stream osteoblast activity instead of reduced fluid flow through the lacuna‐canaliculi system (LCS), which is reported to occur after 12‐months of age (Lai et al. 2015). The enhanced response to exercise when treating with ARC suggests P2Y_2_ inhibition is then increasing the sensitivity to loading or the down‐stream activation of osteoblasts. In either case, this is the first study to demonstrate the potential of inhibiting P2Y_2_ to enhance the mechanotransduction of bone in an aging model.

Gains in the anabolic response to loading following ARC treatment also included a significant reduction in *Sost* and *Tnfsf11* expression. Osteocytes' down‐regulation of *Sost* plays a key role in the mechanotransduction of bone by activating bone formation (Tu et al. 2012; Robling et al. 2008). The underlying mechanisms that suppress *Sost* expression in response to loading are still being established, but is currently understood to be mediated through the initial calcium response to loading through mechanosensitive channels (Lyons et al. 2017). With age the down‐regulation of *Sost* in response to loading is significantly reduced and likely a function of the corresponding loss in calcium signaling (Holguin, Brodt, and Silva 2016; Morrell et al. 2020). In osteoblasts we have found inhibiting P2Y_2_ activation increases the calcium response to repetitive loading (Gardinier et al. 2014). The gains in calcium signaling by inhibiting P2Y_2_ activation are a function of the lack of actin stress fiber formation, which reduces the cellular mechanosensitivity due to changes in cell stiffness (Raqeeb et al. 2011). Targeting other effectors of the P2Y_2_ signaling pathway, namely the activation of LIM Domain Kinase 2 (LIMK2) has also shown to enhance the mechanosensitivity of bone cells (Yang et al. 2015). Thus, the observed gain in Sost down‐regulation during exercise when combined with ARC treatment is considered a function of increased calcium signaling under the repetitive loading of treadmill running.

Unexpectedly, ARC treatment alone produced significant gains in bone formation and bone mass independent of exercise that corresponded with an increase in overall strength. In particular, endocortical bone formation significantly increased with ARC treatment. Given that endocortical bone formation is not characteristic of the response to exercise (Gardinier et al. 2018), the endocortical response to ARC is likely mediated independent of any changes in the mechanosensitivity of bone. Global knockout models of the P2Y_2_ receptor have exhibited an increase in bone mass with that is consistent with our findings when inhibiting P2Y_2_ pharmaceutical (Orriss et al. 2011, 2017). In contrast, a more recent study showed global knockout mice to have impaired bone formation and osteoblast function, leading to an osteopenic phenotype (Xing et al. 2014). In the study by Xing et al., the degree of bone loss varied between 2‐ and 4‐month old mice, while Orriss et al., revealed an increase in trabecular and cortical bone mass starting at 2‐months of age and remained elevated out to 6‐months of age (Orriss et al. 2017). The discrepancy between the studies has been attributed to variations in imaging modalities as well as the background strain of each mouse. In either case, our findings support the hypothesis that P2Y_2_ acts to inhibit bone formation, and that antagonizing P2Y_2_ increases bone formation. Furthermore, antagonizing P2Y_2_ with ARC also lead to gains in tissue strength. In particular, we observed an increase in the pre‐yield work and toughness that suggest a greater energy absorption and resistance to fracture. Although the irregular shape of the tibia presents limitations to estimate tissue properties, the femur lacked adequate space for four‐point bending without inducing shearing between the contact points. As a result, further exploration of tissue properties, such as fracture toughness or fatigue properties, is warranted to fully capture the impact of ARC treatment on tissue properties and the fracture resistance of bone.

Interestingly, culturing primary osteoblasts in the presence of ARC failed to enhance osteoblast mineralization alone, despite gains in endocortical bone formation following ARC treatment. When treating osteoblasts with ATP or UTP, P2Y_2_ activation mitigates mineralization (Hoebertz et al. 2002). Overexpression of P2Y_2_ in primary rat osteoblasts also inhibits mineralization, while collagen deposition remains unchanged (Ellegaard et al. 2017). However, without the addition of nucleotides the beneficial effects of blocking P2Y_2_ function were not evident from our in vitro studies. Instead, we observed a unique shift in the expression of non‐collagenous proteins bone sialoprotein and osteocalcin in the presence of ARC that would likely contribute to the gains in tissue strength. Knockout models of bone sialoprotein present an increase in osteoid at the expanse of mineralized bone, while osteocalcin knockout models present reduced mineral strength due to aberrations in mineral alignment and the mineral‐to‐matrix ratio (Morgan, Poundarik, and Vashishth 2015). Overall, the extent to which variations in bone sialoprotein and osteocalcin expression following ARC treatment improve the tissue properties of bone requires further examination.

Purinergic signaling also plays a key role in osteoclast activation and behavior (Orriss, Burnstock, and Arnett 2010). In our study, ARC treatment of mice significantly reduced trabecular bone loss, suggesting a reduction in osteoclast activity. Although osteoclasts express the P2Y_2_ receptor, directly activating P2Y_2_ in osteoclasts does not contribute to their down‐stream recruitment or activation (Bowler et al. 1998). Consistent with these findings, we found that inhibiting P2Y_2_ with ARC had no effect on osteoclast activation. Instead, ARC had an indirect effect on osteoclast activation that was mediated through a shift in osteocyte communication. Furthermore, the lack of trabecular bone loss in ARC treated mice was associated with a decrease in osteocytes' expression of *Tnfsf11*. Osteocytes' expression and release of *Tnfsf11* is regulated by several mechanisms, such as mechanical loading as well as parathyroid hormone (Xiong and O'Brien 2012). Although our study demonstrates a significant reduction in *Tnfsf11* under exercise, we also found reduced levels independent of any loading, suggesting an alternative mechanism by which P2Y_2_ effects *Tnfsf11* expression that warrants further exploration.

Purinergic signaling through P2Y_2_ is associated with numerous pathologies, such as tumor metastasis, inflammation, atherosclerosis, as well as pulmonary fibrosis (Burnstock 2017). To alleviate such pathologies, the thiouracil derivative AR‐C118925 was recently made commercially available, and has proven to be one of the most selective P2Y2 antagonists (Rafehi et al. 2017). Given the novelty of this drug, few studies have evaluated the utility of AR‐C118925. In mice, Magina et al. found 1 to 7 mg/kg alleviates sub‐chronic trigeminal pain more effectively than other P2Y antagonist (Magni et al. 2015). Administering AR‐C118925 5 days out of the week for up to 10 weeks has also been shown to reduce skin fibrosis as well as suppress pancreatic tumor growth with no adverse side‐effects (Perera et al. 2018; Hu et al. 2018). At a higher dose of 10 mg/kg we were able to demonstrate that ARC may provide a means to preventing bone loss. Although the use of a high dosage demonstrates a proof of concept, a dose response is imperative to fully understand the utility of ARC in preventing bone loss. Furthermore, the off‐targets of ARC must also be considered. In particular, the loss of the P2Y_2_ function in cardiac endothelial cells reduces endothelial nitric oxide synthase (eNOS) activity and production, which can lead to endothelial dysfunction and vasoconstriction (Chen et al. 2017). P2Y_2_ is also considered a key inhibitor of vascular calcification, such that activating P2Y_2_ is being considered a prevention strategy (Qian et al. 2017). In light of these findings, the off‐target effects of AR‐C118925XX must be fully considered in developing clinical strategies targeting P2Y_2_ function, starting with potential changes in blood pressure and overall cardiovascular function.

Given that ARC treatment also produces an anabolic effect independent of loading, ARC treatment may have the potential to prevent other pathologies, such as OVX‐induced bone loss. Estrogen is reported to down‐regulate the expression of P2Y_2_ at both the mRNA and protein level in cancer cell lines, while estrogen deficiency was recently shown to increase P2Y_2_ expression among mesenchymal stem cells (Du et al. 2018; Li et al. 2011). The increase in P2Y_2_ expression was mediated by tumor necrosis factor alpha (TNF‐α) and contributed to the lack of osteoblast function in ovariectomized mice (Du et al. 2018). These findings combined with our study suggest that inhibiting P2Y_2_ with ARC may serve to restore bone formation in models of postmenopausal osteoporosis. Furthermore, the anticipated increase in P2Y_2_ expression due to estrogen deficiency may also contribute to the reported reduction in mechanotransduction at the cellular level and at the tissue level in ovariectomized models (Deepak, Kayastha, and McNamara 2017; Zaman et al. 2006). Overall, the lack of female mice in our study represents a limitation that must be addressed in future work.

Overall, these findings demonstrate that inhibiting P2Y_2_ function has the potential to enhance bone formation, namely the anabolic response to exercise and daily loading. In particular, P2Y_2_ inhibition with AR‐C118925XX enhances osteocytes' down‐regulation of Sost under loading as well as osteoblast activation, while also reducing osteocytes' support of osteoclast activation. Altogether, these findings are significant because they present a novel strategy to enhancing the mechanotransduction of bone in an aging population that is prone to osteoporosis and high fracture risk.

## 4 Materials and Methods

### 4.1 Animal Model

All animal studies were conducted in accordance with the Henry Ford Health Institutional Animal Care and Use Committee. Male 35‐week old C57/BL6J mice were purchased from Jackson Laboratories (Strain #:000664, Bar Harbor, ME). Adult mice were chosen because the response to exercise is minimal (Gardinier et al. 2018), allowing any gains from inhibiting P2Y_2_ to be more evident. At 36‐weeks of age mice were divided into 4 weight‐matched groups: sedentary + vehicle, exercise + vehicle, sedentary + ARC, exercise + ARC. Exercise groups were subjected to 30 min of treadmill running for 5‐days each week. At this speed, C57/Bl6 mice are running between 70% and 80% of their maximal oxygen consumption (VO2max), which is by analogy similar to jogging for humans (Schefer and Talan 1996). Groups treated with ARC (AR‐C118925XX, TORCIS, Minneapolis, MN) were given an intraperitoneal injection (10 mg/kg) each day for 5 weeks. Because the half‐life for ARC is within 3 h (Rafehi et al. 2017), mice were treated no more than 2 h prior to each exercise session. The dosage was selected based on previous work showing 10 mg/kg every 5 days suppress pancreatic tumor growth with no adverse side‐effects (Perera et al. 2018; Hu et al. 2018). A subset of mice were sacrificed immediately after 5 consecutive days of exercise and treatment to isolate mRNA and examine early changes in gene expression at the 1‐week time point. The remaining mice were euthanized after a total of 5‐weeks of exercise and treatment to examine changes in histomorphometry, microarchitecture, and mechanical properties.

### 4.2 Gene Analysis

Trizol extracted mRNA was purified (RNeasy Mini Kit, Qiagen) from tibia samples that had been cleaned of any soft‐tissue and flushed to remove the bone marrow to provide osteocyte‐enriched samples. Complementary DNA (cDNA) (Taqman cDNA Synthesis Kit; Applied Biosystems) was then generated and an Applied BioSystems 7500 RealTime PCR machine was used for real‐time quantitative PCR (qRT‐PCR) along with the respective primers listed in Table S6. All samples were normalized to their respective expression of *Gapdh* using the 2^−ΔΔct^ method.

### 4.3 Histomorphometry

For dynamic histomorphometry, mice received an I.P. injection of alizarin red and calcein at 21 and 3 days prior to euthanasia respectively. Tibia and femur samples were then fixed in paraformaldehyde, dehydrated in graded ethanol, and then embedded in methyl methacrylate (Koldmount, Mager Scientific) for sectioning at the mid‐diaphysis using a diamond wafering blade (Mager Scientific) on a low‐speed saw (South Bay Technology). Sections were polished to ~100 μm thickness and imaged under confocal microscopy to identify the labeled surfaces. ImageJ was then used to quantify mineralizing surfaces (MS/BS), mineral apposition rate (MAR), and bone formation rate (BFR).

### 4.4 Micro‐Computed Tomography (μCT) Imaging and Mechanical Testing

Cortical bone architecture was measured using a custom‐built μCT system as described in our previous work (Gardinier et al. 2018). Both tibia and femur samples were embedded in 1% agarose and scanned with the following settings: 16 μm voxel size, 60 kVp, 0.5 mm aluminum filter, 83 μA, and 720 views over 360°, with each view averaging 4 frames. Images were reconstructed using a greyscale threshold optimized across all the samples. Mechanical testing of the tibia under four‐point bending was then performed using a Mach‐1 Micromechanical System (Biosyntech Canada Inc.). The distance between the outer and inner spans were 9 and 3 mm respectively. Each tibia was loaded with the medial surface under tension until failure at a rate of 0.025 mm/s. Load and displacement were recorded along with the fracture site location. Cortical bone architecture at the fracture site was as well as a standard site midway between the loading points were obtained from the micro‐CT images. The moment of inertia about the anterior–posterior axis (MOI_A/P_) and to the neutral axis from the lateral surface were used to calculate tissue‐level properties as described in our previous work (Gardinier et al. 2018). Femoral trabecular bone morphology was measured beginning at 50 μm proximal of the distal growth plate and extending 10% of the total femoral length toward the diaphysis and excluding cortical bone. Trabecular bone volume fraction, thickness, spacing, and number were calculated using CTAn Skyscan software.

### 4.5 Primary Osteoblast Culture and Functional Assays

Bone marrow stromal cells (BMSCs) were isolated from the femora of C57/BL6J male mice by removing the epiphyses and flushing the bone marrow cavity. The bone marrow was passed through a cell‐strainer and then cultured on type‐1 collagen coated dishes with α‐MEM supplemented with 10% fetal bovine serum (FBS) and 1% penicillin–streptomycin (Sigma St. Louis, MO). After 24 h, the non‐adherent cells were removed and used for osteoclast assays, while the remaining BMSCs were cultured until they reached 80% confluency. For mRNA studies, BMSCs were seeded in 6‐well plates at 1.8 × 10^4^ cells/cm^2^ and cultured with osteogenic inducing media (OIM) containing 100 μM L‐ascorbic acid and 2 mM β‐glycerophosphate (Sigma St. Louis, MO). The mRNA was then extracted after 1 and 7 days using Trizol. For mineralization studies, BMSCs were seeded at the same density in 12‐well plates and cultured for 14 days before staining for Alizarin Red. For experimental treatments, OIM media was supplemented with dimethyl sulfoxide (DMSO) as a vehicle control or AR‐C118925XX at 10 μM. The selected dose for ARC was based on previous in vitro studies showing 10 μM to produce optimal reduction in the response to UTP or ATP (Rafehi et al. 2017).

### 4.6 Primary Osteoclast Culture and Functional Assays

The non‐adherent cells removed during the BMSCs preparation were considered hematopoietic stem cells (HPSCs) and seeded in 48‐well plate at 0.27 × 10^5^ cells/cm^2^. The growth media was supplemented with 50 ng/mL of macrophage colony‐stimulating factor (MCSF, Sigma) and 50 ng/mL of the receptor activator of nuclear factor kappa beta ligand (RANKL, Sigma). For direct effects of ARC, the differentiation media was supplemented with 10 μM AR‐C118925XX or dimethyl sulfoxide (DMSO) as a vehicle control. To examine osteocytes' impact on osteoclast behavior, the differentiation media was supplemented with 10% conditioned media from MLO‐Y4 cells (kindly provided by Lynda Bonewald, Indiana University School of Medicine) treated with ARC (10uM) or DMSO as a vehicle control.

### 4.7 Statistical Analysis

Each experimental group included nine mice. A two‐way analysis of variance (ANOVA) was performed to determine main and interaction effects of treatment (Vehicle vs. ARC) and physical activity (sedentary vs. Exercise) with repeated measures and Tukey post hoc testing between groups. A Student's *t*‐test was used to identify statistical differences between femoral indices for vehicle and ARC sedentary groups. A similar Student's *t*‐test was used to examine outcome measures for all in vitro measurement variables. All in vitro experiments were conducted at least three times, each test consisting of cells from different mice and representing a biological replicate. Throughout the study, a *p* < 0.05 was considered significant.

## Author Contributions

Joseph Gardinier, Amit Chougule, and Chunbin Zhang designed the experiments and analyzed the data. Jordan Denbow, Nickolas Vinokurov, Devin Mendez and Elizabeth Vojtisek preformed analysis of bone microarchitecture. Jordan Denbow and Elizabeth Vojtisek preformed biomechanics analysis. Chunbin Zhang performed animal work. Amit Chougule and Chunbin Zhang, performed in vitro studies. Jordan Denbow performed bone histomorphometry. Joseph Gardinier, Nickolas Vinokurov, and Devin Mendez discussed results. Amit Chougule, Nickolas Vinokurov, Devin Mendez and Joseph Gardinier wrote and revised the manuscript.

## Conflicts of Interest

The authors declare no conflicts of interest.

## Supporting information


Table S1.

Data S1.

## Data Availability

The data are available from the corresponding author upon reasonable request.
