# Supplementary material for: P2Y2 Inhibition Modifies the Anabolic Response to Exercise in Adult Mice
Source: Aging Cell. 2024 Dec 31;24(5):e14464. doi: 10.1111/acel.14464 (PMC12074023; doi:10.1111/acel.14464)
Supplement: Supplementary file 1 — Table S1. [file ACEL-24-e14464-s002.docx]

**Supplementary Materials for**

##

## P2Y2 Inhibition Modifies the Anabolic Response to Exercise in Adult Mice

Amit Chougule^1,2,3^, Chunbin Zhang^1^, Jordan Denbow^1^, Nickolas Vinokurov^4^, Devin Mendez^4^, Elizabeth Vojtisek^1^, Joseph Gardinier^1,2,3^

**Author Affiliation:**

^1^Bone and Joint Center, Henry Ford Health System, Detroit, MI, 48202

^2^Henry Ford Health + Michigan State University Health Sciences, Detroit, MI, 48202

^3^Dept Physiology, College of Human Medicine, Michigan State University, East Lansing, MI, 48824

^4^School of Medicine, Wayne State University, Detroit, MI, 48202

**Corresponding Author:**

Joseph D. Gardinier, Ph.D.

Assistant Scientist

Henry Ford Health + Michigan State University Health Sciences

6135 Woodward Avenue

Detroit, MI 48202

Tel.: +1-313-874-8331

Jgardin2@hfhs.org

**This PDF file includes:**

Table S1: Body weights of mice.

Table S2: Histomorphometry of the tibia following sedentary and exercise conditions.

Table S3: Structural- and tissue-level mechanical properties of the tibia following exercise and sedentary conditions.

Table S4: Histomorphometry of the femur following vehicle or AR-C118925XX treatment.

Table S5: Trabecular bone microarchitecture in the distal femur following vehicle or AR-C118925XX treatment.

Table S6: Mouse PCR primers for mRNA analysis

|  | Body Weight  Mean (std) |
| --- | --- |
| Baseline | 34.1 (3.1) |
| Sedentary | 29.8 (1.9) |
| Exercise | 32.3 (1.7) |
| Sedentary + ARC | 31.8 (1.3) |
| Exercise + ARC | 31.3 (1.2) |

**Supplementary Table 1: Body weights in grams.** Values represent the mean (± std).

|  | Vehicle | | |  | ARC | | Main Effects | | | |  |
| --- | --- | --- | --- | --- | --- | --- | --- | --- | --- | --- | --- |
|  | Sedentary  n=9 | Exercise  n=9 |  | | Sedentary  n=10 | Exercise  n=9 | | T | A | TxA | |
| Ec.MS/BS (µm/µm) | 0.050 (0.029) | 0.066 (0.026) |  | | 0.139 (0.041) * | 0.127 (0.033) * | | **<0.001** | **0.006** |  | |
| Ec.MAR (µm/day) | 0.858 (0.298) | 0.928 (0.495) |  | | 1.560 (0.602) | 1.641 (0.454) | | **<0.001** |  |  | |
| Ps.MS/BS (µm/µm) | 0.054 (0.031) | 0.073 (0.023) |  | | 0.075 (0.052) | 0.123 (0.045) *,ǂ | | **0.012** | **0.015** |  | |
| Ps.MAR (µm/day) | 0.556 (0.224) | 0.694 (0.291) |  | | 0.701 (0.392) | 1.003 (0.355) | |  |  |  | |

**Supplementary Table 2: Histomorphometry of the tibia following sedentary and exercise conditions.**

Two-way ANOVA identified main effects for treatment (T), activity (A) and their interaction (T × A). Tukey post-hoc analysis identified significant differences between groups. Values represent the mean (± std).

* p-value < 0.05 compared to vehicle control

ǂ p-value < 0.05 compared to sedentary control

|  | Vehicle | | |  | ARC | | | | Main Effects | | | | | |  |
| --- | --- | --- | --- | --- | --- | --- | --- | --- | --- | --- | --- | --- | --- | --- | --- |
|  | Sedentary  n=9 | Exercise  n=9 | |  | Sedentary  n=10 | | Exercise  n=9 | | T | | A | | TxA | |  |
| **Structural-Level** |  | |  | |  |  | |  | |  | |  | |  | |
| Yield  Displacement (µm) | 196.6 (43.1) | | 211.9 (72.6) | |  | 235.2 (25.6) * | | 244.103 (47.48) | | **0.049** | |  | |  | |
| Ultimate Displacement (µm) | 292.9 (64.7) | | 285.7 (61.3) | |  | 284.4 (62.1) | | 277.4 (53.2) | |  | |  | |  | |
| Post-Yield Displacement (µm) | 201.89 (111.5) | | 235.5 (143.3) | |  | 439.5 (496.8) | | 222.6 (231.3) | |  | |  | |  | |
| Yield Load (N) | 11.20 (2.70) | | 12.01 (3.26) | |  | 17.27 (2.37) | | 17.08 (1.73) | | **<0.001** | |  | |  | |
| Ultimate Load (N) | 12.79 (2.39) | | 13.88 (2.63) | |  | 18.31 (2.17) * | | 17.72 (2.07) * | | **<0.001** | |  | |  | |
|  |  | |  | |  |  | |  | |  | |  | |  | |
| **Tissue-Level** |  | |  | |  |  | |  | |  | |  | |  | |
| Yield Strain (µε) | 18128 (3534) | | 21327 (8007) | |  | 19575 (4251) | | 18397 (3153) | |  | |  | |  | |
| Ultimate Strain (µε) | 27190 (6163) | | 28733 (6820) | |  | 23583 (6140) | | 20935 (3836) * | | **0.009** | |  | |  | |
| Yield Stress (MPa) | 113.1 (33.5) | | 97.8 (40.3) | |  | 143.2 (19.7) * | | 131.6 (13.7) * | | **<0.001** | | **0.0123** | |  | |
| Ultimate Stress (MPa) | 128.5 (30.8) | | 112.6 (37.0) | |  | 151.8 (18.5) | | 136.4 (14.1) | | **<0.001** | | **0.002** | |  | |

**Supplementary Table 3: Structural- and tissue-level mechanical properties of the tibia following exercise and sedentary conditions.** Two-way ANOVA identified main effects for treatment (T), activity (A) and their interaction (T × A). Tukey post-hoc analysis identified significant differences between groups. Values represent the mean (± std).

* p-value < 0.05 compared to vehicle controls.

|  | Vehicle  n=9  mean (± std) | ARC  n=10  mean (± std) |  |
| --- | --- | --- | --- |
| Ec.MS/BS (µm/µm) | 0.088 (0.030) | 0.209 (0.052) ** |  |
| Ec.MAR (µm/day) | 1.763 (0.268) | 1.713 (0.660) |  |
| Ps.MS/BS (µm/µm) | 0.120 (0.057) | 0.168 (0.076) |  |
| Ps.MAR (µm/day) | 1.087 (0.315) | 0.836 (0.373) |  |

**Supplementary Table 4: Histomorphometry of the femur following vehicle or AR-C118925XX treatment.** Student t-test identified significant differences between groups. Values represent the mean (± std).

** p-value < 0.001 compared to vehicle treatment.

|  | Baseline  n=9 | Vehicle  n=9 | ARC  n=10 |  |
| --- | --- | --- | --- | --- |
| SMI (A.U.) | 2.28 (0.34) | 2.19 (0.12) | 1.99 (0.15) * |  |
| Degree of Anisotropy (A.U.) | 1.49 (0.19) | 1.33 (0.14) ǂ | 1.55 (0.15) * |  |
| Connectivity Density (mm^-3^) | 188.6 (105.8) | 95.5 (39.7) ǂ | 176.2 (39.5) * |  |

**Supplementary Table 5: Trabecular bone microarchitecture in the distal femur following vehicle or AR-C118925XX treatment.** Student t-test identified significant differences between groups. Values represent the mean (± std).

* p-value < 0.01 compared to vehicle treatment.

ǂ p-value < 0.05 compared to baseline.

| **Gene** | **Forward** | **Reverse** |
| --- | --- | --- |
| Gapdh | CATCACTGCCACCCAGAAGACTG | ATGCCAGTGAGCTTCCCGTTCAG |
| Rankl (Tnfsf11) | GCTGGGCCAAGATCTCTAAC | GTAGGTACGCTTCCCGATGT |
| Opg (Tnfrsf11b) | ACCCAGAAACTGGTCATCAGC | CTGCAATACACACACTCATCACT |
| Sost | CTTCAGGAATGATGCCACAGAGGT | ATCTTTGGCGTCATAGGGATGGTG |
| Collagen-1a (Col1a1) | GCTCCTCTTAGGGGGCACT | CCACGTCTCACCATTGGGG |
| Osteocalcin (Bglap) | CTTGGGTTCTGACTGGGTGT | TGGCCACTTACCCAAGGTAG |
| Osteopontin (Opn) | TGCACCCAGATCCTATAGCC | CTCCATCGTCATCATCATCG |
| Osteonectin (Ocn) | CCTCTAAACCCCTCCACATTCCT | GCCAGGCAAAGGAGAAAGAAGAT |
| Bone Sialoprotein-2 (Bsp) | GAGACGGCGATAGTTCC | AGTGCCGCTAACTCAA |
| Alkaline phosphate (Alp) | ACACCTTGACTGTGGTTACTGCTG | CCTTGTAGCCAGGCCCGTTA |

**Supplementary Table 6: Mouse PCR primers for mRNA analysis.**
