# Supplementary material for: P2Y2 Inhibition Modifies the Anabolic Response to Exercise in Adult Mice
Source: Aging Cell. 2024 Dec 31;24(5):e14464. doi: 10.1111/acel.14464 (PMC12074023; doi:10.1111/acel.14464)
Supplement: Supplementary file 2 — Data S1. [file ACEL-24-e14464-s001.pdf]

AGING CELL AUTHOR CHECKLIST. ***Authors should submit this checklist together with their manuscript. Please ensure that you have read the Author Guidelines in detail before submission.***

**Title**	P2Y2 Inhibition Modifies the Anabolic Response to Exercise in Adult Mice						
**Authors**	Amit Chougule, Chunbin Zhang, Jordan Denbow, Nickolas Vinokurov, Devin Mendez, Elizabeth Vojtisek, Joseph Gardinier						
**Manuscript Type**	Primary Research Paper						
**Total Character Count (including spaces)[1]**	39,053						
**Word count of Summary[2]**	236						
**Number of papers cited in the References[3]**	45						
**Listing of all Tables (Table1, Table 2 etc)[4]**	0						
**Figure specifications (please complete one row per figure)[5]** ***Figure no.***	Colour ***(yes/no)***	Greyscale ***(yes/no)***	Black and white ***(yes/no)***	Single column (80mm) ***(yes/no)***	Double column (180mm) ***(yes/no)***	Size of figure at full scale (mm x mm) ***(insert details)***	Smallest font size used in the figure at full scale (minimum 6pt) ***(insert details)***
1	yes				yes	180 x 150	7pt
2	yes				yes	180 x 142	7pt
3	yes				yes	180 x 164	7pt
4	yes				yes	180 x 131	7pt
5	yes			yes		80 x 134	7pt

[1] The maximum character count allowed is 50,000 (incl. spaces) for Primary Research Papers and Reviews, 10,000 for Short Takes.

[2] Summary should not exceed 250 words.

[3] Primary Research Papers can contain a maximum of two tables. If more are needed they should replace some of the Figures or can be placed in the Supporting Information.

[4] A maximum of 45 references is allowed for Primary Research Papers and 20 references for Short Takes.

[5] A Primary Research Paper may contain up to 6 figures and a Short Take up to 2 figures. Authors are encouraged to provide figures in the size they are to appear in the journal and at the specifications given.
